# Complete plastid genome of *Rhododendron griersonianum*, a critically endangered plant with extremely small populations (PSESP) from southwest China

**DOI:** 10.1080/23802359.2020.1800427

**Published:** 2020-07-30

**Authors:** Detuan Liu, Chaonan Fu, Lingjuan Yin, Yongpeng Ma

**Affiliations:** a Yunnan Key Laboratory for Integrative Conservation of Plant Species with Extremely Small Populations, Kunming Institute of Botany, Chinese Academy of Sciences, Kunming, China; b Key Laboratory for Plant Diversity and Biogeography of East Asia, Chinese Academy of Sciences, Kunming, China; c Lijiang National Secondary Professional School, Lijiang, China

**Keywords:** *Rhododendron griersonianum*, PSESP, Plastid, Phylogeny

## Abstract

The complete plastid genome of *Rhododendron griersonianum*, a critically endangered plant species with extremely small populations, was obtained using Illumina HiSeq X Ten and ONT PromethION sequencing. The full length of the plastid genome is 206,467 bp with an overall GC content of 35.8%, which encodes 118 unique genes, including 78 protein-coding genes, 36 tRNA and 4 rRNA genes. Phylogenetic analysis revealed that all *Rhododendron* species formed a monophyletic clade. This study provides a valuable reference and will facilitate future studies related to the general characteristics and evolution of plastid genomes in the genus *Rhododendron*.

The genus *Rhododendron* L. belongs to the family Ericaceae and contains more than 1,000 species distributed worldwide (Chamberlain et al. 1996). *R. griersonianum* Balf. f. et Forrest is a critically endangered (CR) species listed both in *The Red List of Rhododendrons* (Gibbs et al. 2011) and in the *Threatened Species List of China’s Higher Plants* (Qin et al. 2017). It has been reported that no more than 350 individuals have been found in the wild (Liu et al. 2019). In the present study, plastid genome of *R. griersonianum* was generated using Illumina Novaseq and ONT PromethION sequencing platforms.

Genomic DNA was extracted from young leaves collected from a single wild individual of *R. griersonianum* in Tengchong City, Yunnan Province of China (98°39′56.53″E, 25°33′24.81″N). And the specimens had been submitted to the Germplasm Bank of Wild Species, Southwest China (collection number: MYP20191001). Genome sequencing was performed on Illumina HiSeq X Ten and ONT PromethION sequencing platforms. GetOrganelle v1.7.0 (Jin et al. 2018), SMARTdenovo (Ruan 2018) and fmlrc (Wang et al. 2018) were used to assemble, polish and correct the genome. Annotation was performed using PGA (Qu et al. 2019), then manually corrected with the plastid genome *R. delavayi* (Liu et al. 2020) as reference. The final length of the plastid genome is 206,467 bp, which encodes 118 unique genes, including 78 protein-coding genes, 36 tRNA and 4 rRNA genes, with a GC content of 35.8%, and comprising of a SSC (small single copy) region of 315 bp, a LSC (large single copy) region of 111,218 bp, and a pair of IR regions of 94,934 bp. A total of 50 pairs of long repeat sequences with length of 144 bp to 802 bp were identified in the whole plastid genome by the web service of REPuter (Kurtz and Schleiermacher 1999). The newly annotated complete plastid genome was submitted to GenBank, where the received accession number was MT533181 and had been released.

To study the phylogenetic position of *R. griersonianum*, we downloaded the whole chloroplast genomes of 13 species from NCBI database (https://www.ncbi.nlm.nih.gov). The protein-coding genes were extracted, concatenated using Phylosuite v1.2.1 (Zhang et al., 2020) and then used to make a NJ trees (Figure 1).

**Figure 1.**
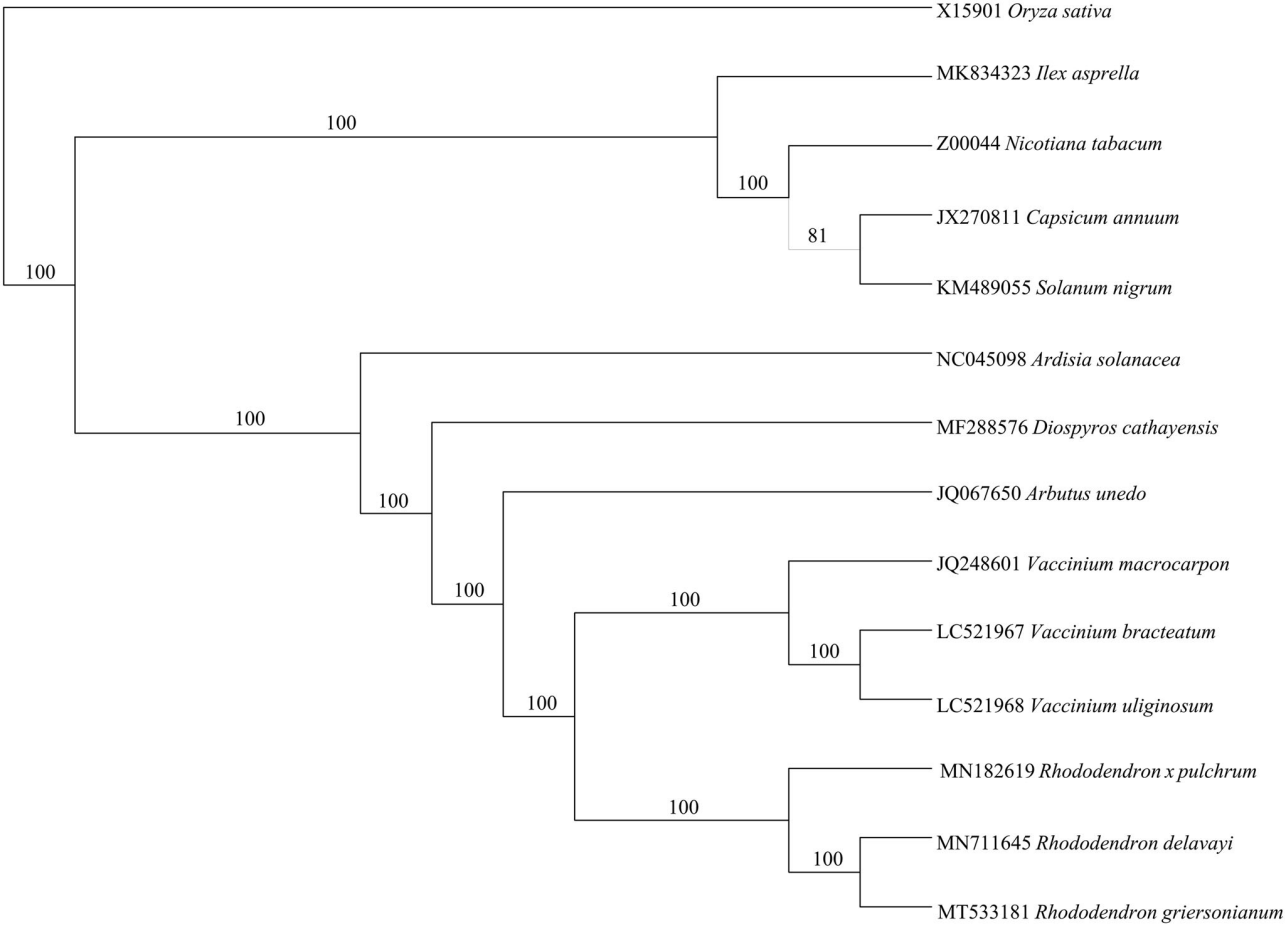
Phylogenetic analysis based on protein-coding genes in plastid genomes by Neighbor-Joining with 1,000 replicates and *Oryza sativa* as out group.

Our study represents the first look into the complete plastid genome of *R. griersonianum*, which is the third plastid genome in genus *Rhododendron,* with 12,669 bp longer than *Rhododendron delavayi* (Liu et al. 2020) and 70,218 bp longer than *Rhododendron* × *pulchrum* (Shen et al. 2019). The availability of *R. griersonianum* plastid genome will greatly contribute to the evolutionary and phylogenetic analysis, and will benefit future studies related to the development and horticultural utilization in the genus *Rhododendron*.

## Data Availability

The data that support the findings of this study are also accessible in figshare at https://doi.org/10.6084/m9.figshare.12616781

## References

[CIT0001] Chamberlain D, Hyam R, Argent G, Fairweather G, Walter KS. 1996. The genus Rhododendron: its classification and synonymy. Edinburgh: Royal Botanic Garden.

[CIT0002] Gibbs D, Chamberlain D, Argent G. 2011. The red list of rhododendrons. Botanic Gardens Conservation International.

[CIT0003] Jin JJ, Yu WB, Yang JB, Song Y, Yi TS, Li DZ. 2018. GetOrganelle: a simple and fast pipeline for de novo assembly of a complete circular chloroplast genome using genome skimming data. BioRxiv. e256479.

[CIT0004] Kurtz S, Schleiermacher C. 1999. REPuter: fast computation of maximal repeats in complete genomes. Bioinformatics. 15(5):426–427. 10366664 10.1093/bioinformatics/15.5.426

[CIT0005] Liu D, Sun W, Ma Y, Fang Z. 2019. Rediscovery and conservation of the Critically Endangered *Rhododendron griersonianum* in Yunnan, China. Oryx. 53(1):14–14.

[CIT0006] Liu J, Chen T, Zhang Y, Li Y, Gong J, Yi Y. 2020. The complete chloroplast genome of *Rhododendron delavayi* (Ericaceae). Mitochondrial DNA Part B. 5(1):37–38. 10.1080/23802359.2019.1689860 PMC7721020 33366411

[CIT0007] Qin H, Yang Y, Dong S, He Q, Jia Y, Zhao L, Yu S, Liu H, Liu B, Yan Y, et al. 2017. Threatened species list of China’s higher plants. Biodiversity science. 25(7):696–744.

[CIT0008] Qu X-J, Moore MJ, Li DZ, Yi TS. 2019. PGA: a software package for rapid, accurate, and flexible batch annotation of plastomes. Plant Methods. 15:50. 31139240 10.1186/s13007-019-0435-7 PMC6528300

[CIT0009] Ruan J. 2018. SMARTdenovo: ultra-fast de novo assembler using long noisy reads. [Accessed 2020 Feb 10]. https://github.com/ruanjue/smartdenovo. 10.46471/gigabyte.15 PMC9632051 36824332

[CIT0010] Shen JS, Li XQ, Zhu XT, Huang XL, Jin SH. 2019. Complete chloroplast genome of *Rhododendron pulchrum*, an ornamental medicinal and food tree. Mitochondrial DNA Part B. 4(2):3527–3528. 33366070 10.1080/23802359.2019.1676181 PMC7707349

[CIT0011] Wang JR, Holt J, McMillan L, Jones CD. 2018. FMLRC: hybrid long read error correction using an FM-index. BMC bioinformatics. 19(1):50. 29426289 10.1186/s12859-018-2051-3 PMC5807796

[CIT0012] Zhang D, Gao F, Jakovlić I, Zou H, Zhang J, Li WX, Wang GT. 2020. PhyloSuite: an integrated and scalable desktop platform for streamlined molecular sequence data management and evolutionary phylogenetics studies. Molecular Ecology Resources. 20(1):348–355. 31599058 10.1111/1755-0998.13096

